# New avatars for Myriapods: Complete 3D morphology of type specimens transcends conventional species description (Myriapoda, Chilopoda)

**DOI:** 10.1371/journal.pone.0200158

**Published:** 2018-07-03

**Authors:** Nesrine Akkari, Anne-Sarah Ganske, Ana Komerički, Brian Metscher

**Affiliations:** 1 3rd Zoological Department, Natural History Museum Vienna, Vienna, Austria; 2 Department of Integrative Zoology, University of Vienna, Vienna, Austria; 3 Croatian Biospeleological Society, Zagreb, Croatia; 4 Department of Theoretical Biology, University of Vienna, Vienna, Austria; Monash University, AUSTRALIA

## Abstract

We present high-resolution X-ray microtomography (microCT) to enhance the standard morphological description of a recently described centipede, *Eupolybothrus liburnicus* Akkari, Komerički, Weigand, Edgecombe and Stoev, 2017. The 3D images of the holotype and paratype specimens are considered here as cybertypes for the species–a universal and virtual representation of the type material. This ‘avatar’ of the holotype is the first published male centipede cybertype. The microtomographic data of both types revealed further characters of systematic value and allowed us to hypothesise on the function of some of the male secondary structures and the mating behaviour of the species. Additionally, we compared part of the female reproductive system of *E*. *liburnicus* to species from the same genus, including *E*. *cavernicolus* Stoev & Komerički 2013, its closest congener. The high-resolution 3D image data have been uploaded to an open repository (MorphoSource.org) to serve in any subsequent study on the species and genus, as we believe this would catalyse biosystematic research on this and other arthropod groups.

## Introduction

Even in the era of genomic-dominated approaches in systematics, the need for morphology has remained essential to understand the organismal diversity and relationships (e.g. [1], [2], [3], [4] and references therein). From the basic fact of describing taxa to the study of fossils to calibrate phylogenetic trees, understand the evolutionary histories and interrelationships of taxa or comparing structures to understand their function, retracing the ontogeny of organisms and their development, or even to analyse all these aspects in an integrative way, the study of morphology has remained universal and essential. A large array of techniques exploring surface and internal structures of invertebrates is already available, and new methods continue to emerge; see those reviewed in [5]. These methods, combined with the traditional ones, have tremendously enhanced the amount and quality of morphological information obtained from diverse organisms.

The use of X-ray microtomography (microCT) was one of the methods that recently catalysed morphology-based research in biology. Combined with advanced software, microCT has enabled the study of soft tissues, musculature and other internal structures [4]. Similarly, microCT has gained increasing popularity in modern systematics of Arthropods and other organisms [1] and examples therein. The use of this technique in the biosystematics of Myriapoda was tentatively applied to the study of amber fossils [6] and references therein. On the other hand, Akkari et al. created the first cybertype millipede, *Ommatoiulus avatar* Akkari and Enghoff, 2015 [1], while Stoev et al. included microCT images of the centipede *Eupolybothrus cavernicolus* (the "cybercentipede of the future") in their holistic description of the species [7] with the volumetric data published in [8].

In this paper, we further promote the creation and use of ‘myriapod avatars’ and present volumetric image data of the male holotype and female paratype of *Eupolybothrus liburnicus*, a recently described cave centipede and the closest congener to the hitherto published cybercentipede *E*. *cavernicolus*. The avatars of *E*. *liburnicus* include complete 3D image datasets of the whole holotype and one female paratype, and high-detail images of the head and ultimate segments. The microCT data is the only available source of information on further structures with potential biological and systematic value and is necessary to complement the conventional morphological and molecular characterisation of the species as presented in [9].

## Material and methods

The male holotype and a female paratype, originally preserved in 70% ethanol and kept in the collections of the Croatian Biospeleological Society (CBSS), were for this study, fixed in alcoholic Bouin's (equal parts Bouin's solution and absolute ethanol) overnight and changed back to 70% ethanol. They were then dehydrated to absolute ethanol (96–100%), stained with 1% (w/v) iodine in ethanol (I2E) for 1–2 days [10], and changed back to ethanol. For stabilisation of the samples during scanning, they were mounted in agarose (0.5–1.0% aqueous) in plastic tubes (5000 μl micropipette tips) [11]. The X-ray image contrast was less than desired, so the samples were rehydrated and stained in IKI [10] overnight, a procedure that has given favourable results with other arthropod specimens (unpublished).

Both specimens were imaged as series of partial scans which were stitched together after tomographic reconstruction, and higher-resolution scans were made of the head and posterior regions (in particular the prefemora of the male). The overview image of the female paratype was made with a SkyScan 1174 (Bruker microCT; tungsten source, 50 kVp, 40 W) and reconstructed with 18.3 μm voxels. The overview image of the male holotype and high detail images of the anterior and posterior parts of the specimen were made with an Xradia MicroXCT-200 system (Zeiss Microscopy) equipped with a micro-focus tungsten source (Hamamatsu L9421-02) and switchable scintillator-objective lenses. Images were made with source voltage of 80 kVp, no beam filter, and source-object-detector distances adjusted to give the desired field view for each sample. The acquisition and reconstruction parameters for each scan are detailed in the metadata files archived with the cybertype image data. Projection images were taken every 0.2° over a half-rotation (180° plus the beam cone angle; [11]). After imaging, the agarose was removed manually using 6M KI and a paintbrush and blunt wooden applicator stick. Samples were then returned to 70% ethanol, which washed out the iodine.

Tomographic sections were reconstructed using the Xradia software (XMRecontructor, v. 8.1) or Bruker NRecon v. 1.7 with pixel sizes and slice thicknesses of 2.7–18.3 μm (isotropic voxels), depending on the field of view. The window and level values for the images were chosen in the reconstruction to include the complete brightness range of the images. Diffusion of iodine into the mounting medium (aqueous agarose) resulted in a discernible gradient of background absorption from one end of the sample to the other. This was largely corrected by adjusting the beam-hardening parameter during reconstruction.

The reconstructed tomographic images were exported as 8-bit TIFF image stacks (no compression and retaining the original pixel values), and reoriented, cropped and resliced parasagittally using Fiji [12], then saved as grayscale TIFF image stacks. For archiving, the stacks were reoriented and cropped in Fiji.

The taxonomically important structures were segmented in Amira 6.3 and 6.4 and highlighted in the volume renderings using selected colormaps and adjusted opacities (not as surface renderings, which eliminate much of the significant 3D information). Scans of *E*. *cavernicolus* were downloaded from GigaDB repository [8] and segmented in Amira 6.0.1. All videos were made in Amira 6.3 and 6.4 software and compiled with Windows Movie Maker or Apple QuickTime Player 10.4.

Figures were processed in Adobe Photoshop CS6 and assembled in Adobe InDesign CS6.

No permits were required for the described study.

## Results

### Volumetric data and 3D reconstructions of the cybertypes

#### Cybertypes

Volume image data of the physical types: Holotype, Ad. ♂, Croatia, Obrovac, Gornji Čabrići, cave Plitka peć, under rock, 09.II.2013, A. Komerički leg., CBSS–CHP 545; Paratype, Ad. ♀, Croatia, Obrovac, Gornji Čabrići, cave Plitka peć, 23.VI.2012, T. Vujnović, A. Komerički & R. Baković leg., CBSS –CHP 538. Contrast-enhanced microCT images with 2.7–18.3 μm voxel sizes, archived as grayscale TIFF image stacks.

All 3D images and metadata of the physical types are deposited at MorphoSource.org, where they can be freely accessed as a universal representation of the physical type material for any subsequent morphological and biosystematic studies.

The direct links to the online image data are as follows:

The project "*Eupolybothrus* cybertypes:"

http://www.morphosource.org/Detail/ProjectDetail/Show/project_id/449

Cybertype CBSS-CHP 545: http://www.morphosource.org/Detail/MediaDetail/Show/media_id/21371

Cybertype CBSS-CHP 538:

http://www.morphosource.org/Detail/MediaDetail/Show/media_id/21365

### New characters revealed with microCT

#### Male sexual characters

**Fig 1:**
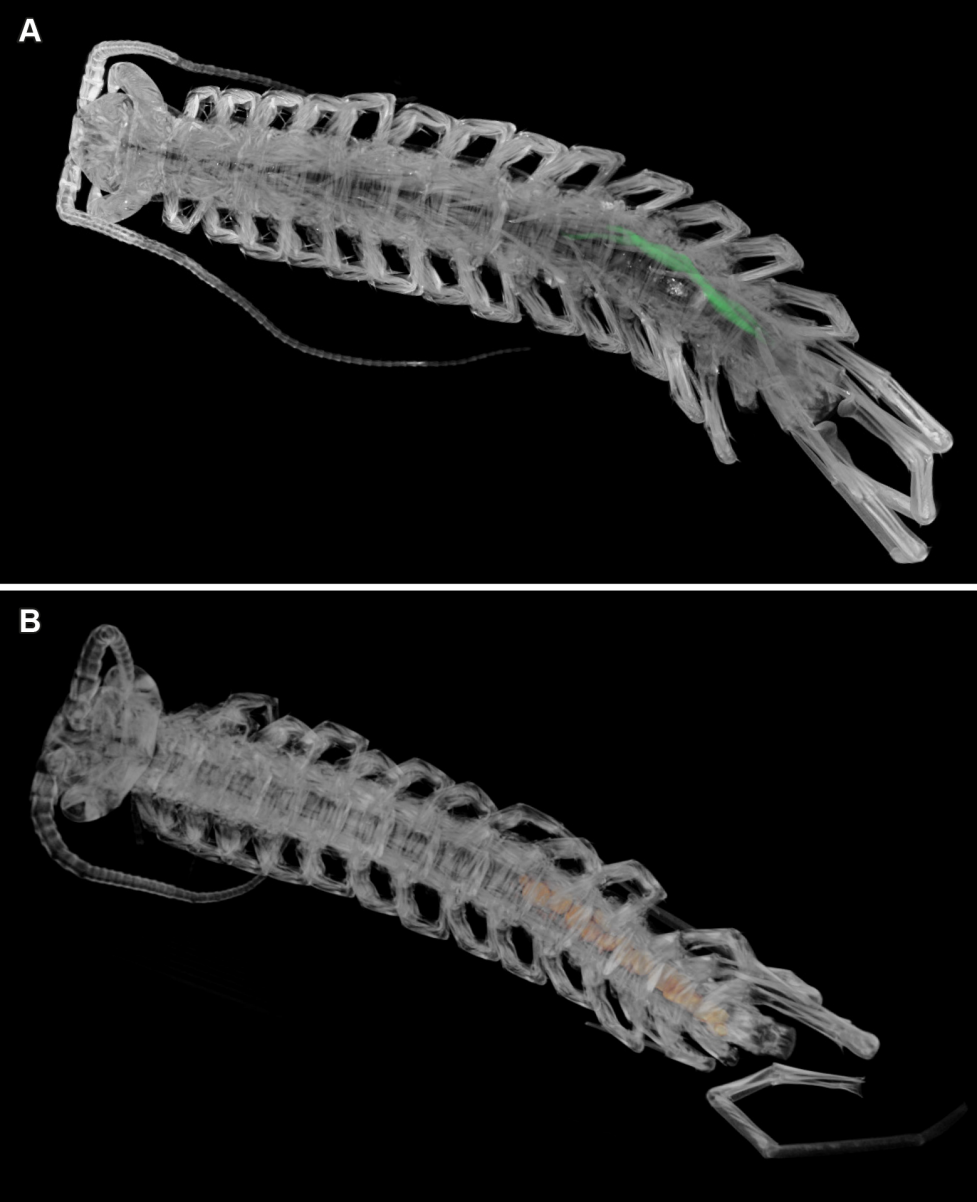
*Eupolybothrus liburnicus*, volume renderings of habitus highlighting selected parts of the reproductive system. **A** Male holotype CHP 545, seminal vesicles (green), dorsal view. **B** Female paratype CHP 583, eggs (orange), ventral view.

**Fig 2:**
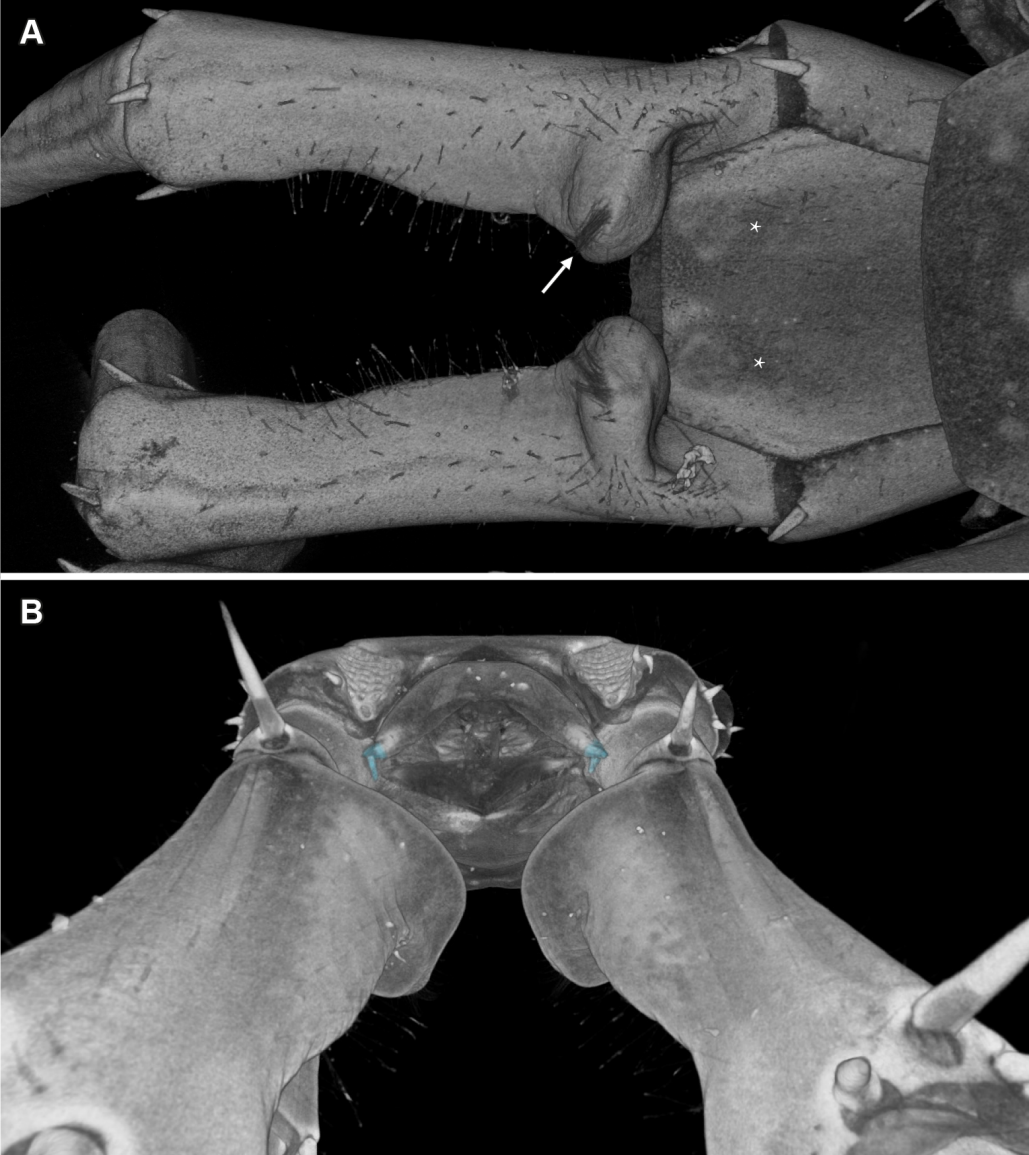
*Eupolybothrus liburnicus*, volume renderings of male holotype CHP 545, intermediate tergite, genital segment and prefemora of legs 15 with knobs. **A** Prefemoral knobs with cluster of setae (*pk*) and intermediate tergite with setae free areas (*sfa*), dorso-lateral view, right is anterior. **B** Prefemoral knobs and genital segment with gonopods (cyan), posterior view, top is ventral.

**Fig 3:**
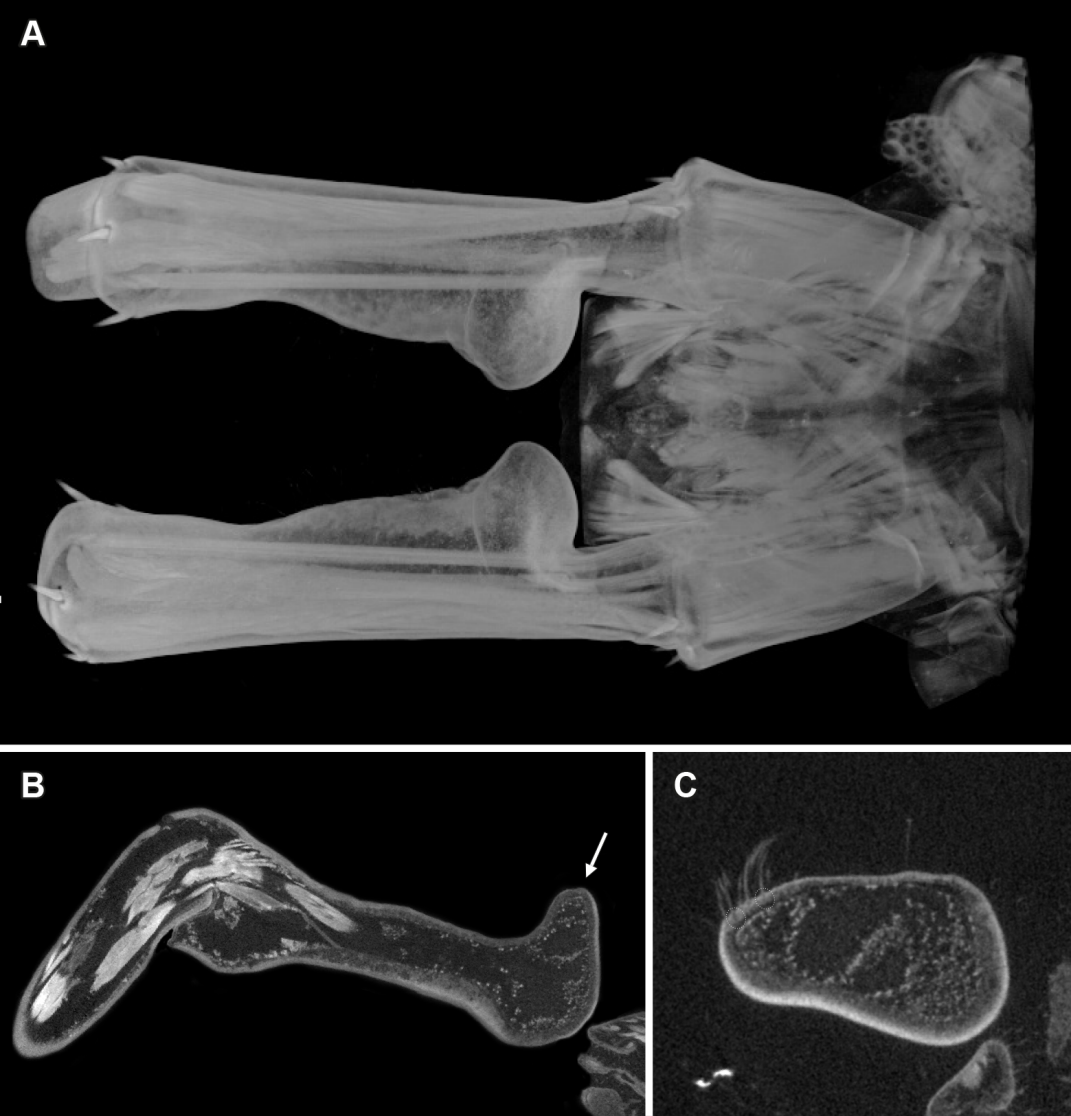
*Eupolybothrus liburnicus*, volume rendering and virtual sections of male holotype CHP 545, intermediate tergite and prefemora of legs 15. **A** Prefemoral knobs, intermediate tergite, musculature, dorsal view, right is anterior. **B** Parasagittal section of right prefemur and prefemoral knob showing musculature and inner structure, top is dorsal. **C** Parasagittal section of prefemoral knob with detail of cluster of setae and cuticular breakthroughs (dashed circles), top is dorsal. Arrow indicating the prefemoral knob.

The scans of the male holotype (Fig 1A) allowed us to depict and reconstruct the male gonopods (Fig 2B), concealed above the genital sternite in this holotype specimen, which are otherwise often hidden and inaccessible for study without performing a dissection (see [9]). Additionally, we had a closer look at the morphology of the prefemoral knobs, a major secondary sexual character in this species (Figs 2 and 3) and present in several species of the genus *Eupolybothrus* Verhoeff, 1907. When present, they display different shapes, bear a number of structures useful for species identification and are presumed to have a role in the reproduction of the species. The complexity of the prefemoral knobs was indicated by conventional representations, but their three-dimensional aspect is clearly portrayed here. In fact, these structures protrude not only medially as described earlier but also dorsally where the cluster of setae is attached (Figs 2 and 3A).

The volumetric data obtained for the male holotype of *E*. *liburnicus* further depict an internal connection of the proximal knob with a hollow area occupying 2/3 of the median section of the prefemur (Fig 3A). The contrast medium highlights the various muscles longitudinally crossing the prefemur but also displays the inner part of the knob and the pit as a highly dense area, spongy and diffuse (Fig 3A, 3B and 3C).

The setae forming the cluster on the apical extremity of the knob are here presumed to be sensory setae or epidermal sensilla. In fact, the base of these setae penetrates the cuticle (Fig 3C) making the sensillum lumen very likely in contact with the sensory cells embedded in the epidermis. However, this needs to be verified with ultrastructural investigations.

The seminal vesicles (Fig 1A) are highlighted in the volume rendering of the holotype to constitute the first documentation of this part of the male reproductive system of the species.

#### Female sexual characters

**Fig 4:**
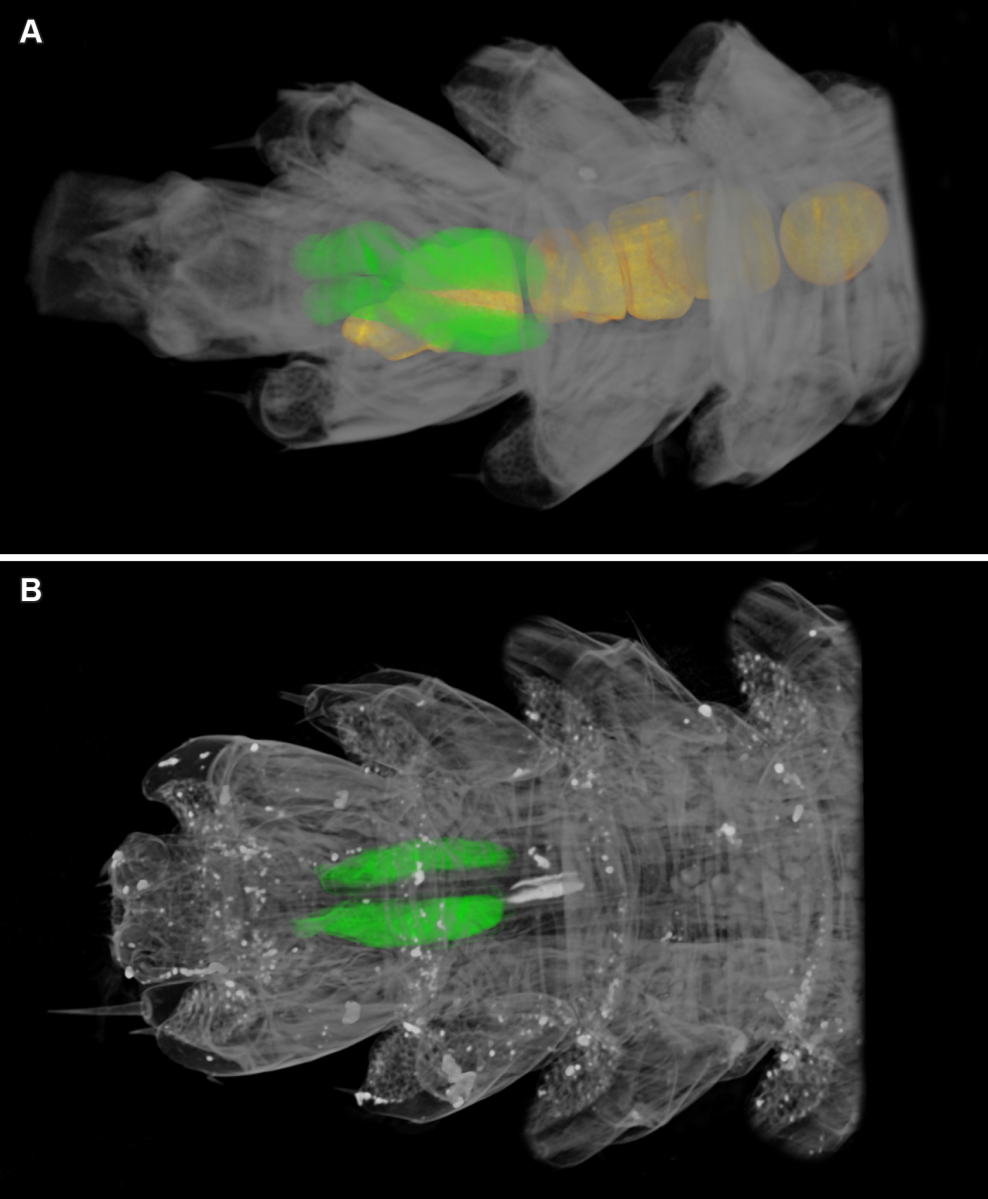
Volume renderings of posterior parts of female *Eupolybothrus*. **A** *Eupolybothrus liburnicus*, paratype CHP 583, highlighted eggs (orange) and seminal receptacles (green). **B** *Eupolybothrus cavernicolus*, highlighted seminal receptacles (green). Ventral view, right is anterior.

Eggs and seminal receptacles are segmented to present the first volumetric information on the female reproductive system of the species (Figs 1B and 4A, S2 Video). The seminal receptacles of *E*. *liburnicus* are sausage-shaped (Fig 4A) and found to be similar to those of *E*. *cavernicolus*, its closest congener (Fig 4B). These structures are however different from the ovoid-shaped receptacles of *E*. *transsylvanicus* (Latzel, 1882) as described by Prunescu [13]. This information is perhaps of potential systematic value as both *E*. *cavernicolus* and *E*. *liburnicus* are currently placed in the same subgenus or species group, *Schizopolybothrus* Verhoeff, 1934 whereas *E*. *transsylvanicus* is placed in the subgenus *Mesobothrus* Verhoeff, 1937.

## Discussion

### Insights on the biology and reproduction of Lithobiomorpha

Information on the biology and reproduction of Lithobiomorpha is scarce, although sexual dimorphism has been documented in a number of species of the class Chilopoda and the modifications of the ultimate legs were recently reviewed [14]. Kenning et al. further summarised the reports on the possible role of these appendages in preying, mating, and defensive strategies and as sensory organs [14].

In the order Lithobiomorpha, the modifications of the ultimate legs are described for many species, as they bear taxonomically important characters used to differentiate the species. Males of the genus *Eupolybothrus* have been documented on a number of occasions as pointing to sexual dimorphism and in taxonomic treatments (see [15], [16], [17], [7], [9]). In fact, various modifications of ultimate legs of males have so far been recorded, including a proximal prefemoral knob surmounted by setae; a distal prefemoral circular protuberance; dorsal sulci on prefemur, femur and tibia; a femoral tuft of setae; a basal femoral pit; spinous setae and a pore-free area. These are considered as important taxonomic characters, although their presence as well as variation, have not been comparatively analysed in all species of the genus, but were mostly compared within species of the same species groups or subgenera. This has resulted in a lack of knowledge on their diversity and physiological function though they are considered important in recognizing potential mates during courtship.

The prefemur of the ultimate legs of *E*. *liburnicus* exhibits special structures, e.g. a proximal prefemoral knob surmounted by a cluster of long setae located dorsally. A close examination of these prefemoral setae (Fig 5B in [9]) inspired us to compare them with the sensilla trichodea described on the antenna of various centipede species, i.e. *Lithobius forficatus* (Linnaeus, 1758), *Scutigera coleoptrata* (Linnaeus, 1758) and *Cryptops hortensis* (Donovan, 1810) ([18], [19], [20], respectively). These sensilla are generally assumed to have a mechano- and chemoreceptive function (and are named as such), a role very plausibly also played by the prefemoral ones. This comes as no surprise as several cuticular sensory microstructures of the ultimate legs have hitherto been homologised to those of the antenna ([14] and references therein).

The mating behaviour and sperm transfer of Lithobiomorph centipedes has been studied and the courtship of *Lithobius forficatus* described in detail [21], revealing a rather long process lasting more than 4 hours and involving a complex exchange of chemo-/mechano-sensorial signals between the partners via the antennae and ultimate legs. During the last stage of the mating ritual, the female brings her ultimate legs on the dorsal side of the male's posterior end to collect the spermatophore, which the male just released and placed on the web between his ultimate legs. Here, we hypothesise that the cluster of sensory setae or ‘sensilla’ on the proximal prefemur observed in *E*. *liburnicus* (perhaps as well as the other prefemoral setae) are responsible for conveying a mechano-sensory information about the female's correct position and readiness before the male releases the spermatophore. This would be to ensure the success of sperm transfer. It remains to be investigated, however, how this information is conveyed and how the various structures of the ultimate legs contribute to the success of the mating in the species without prefemoral knobs.

### Staining techniques for myriapods and other museum zoological specimens

Another centipede cybertype (*E*. *cavernicolus*) was published recently from a morphologically similar and genetically close congener of *E*. *liburnicus* [8]. The microCT information in that report was based on a single dataset of a female paratype, which was desiccated with hexamethyldisilazane (HMDS), resulting in evident shrinkage of the animal, and scanned with 8 μm voxel size with no further contrast enhancement. This definitely affects the quality of the obtained data (see also [22]) which was nonetheless useful to compare both types and species. In fact, we retrieved those data here to reconstruct and compare the seminal receptacles in both female paratypes, and this further confirmed the resemblance between the two species.

Here, we include for *E*. *liburnicus* contrast-enhanced microCT images of the male, as the first centipede cyber-holotype to be published, and we give high-detail images of the posterior segments and ultimate legs, focusing on the prefemur. The microCT data are also currently contributing to another ongoing study of the genus *Lithobius* Leach, 1814 in a comparative approach targeting internal head structures and the reproductive system.

Museum specimens, especially arthropods, are commonly preserved in 70–80% ethanol, frequently without other tissue fixation. Procedures for refixing and staining such samples for X-ray imaging have never been systematically optimised, but our recent experience has shown that refixing with alcoholic Bouin's followed by staining with alcoholic iodine (I2E) can give good tissue preservation and favourable X-ray contrast [10]. However, samples in absolute alcohol can present problems when mounted in aqueous agarose, particularly due to the formation of bubbles. In some cases, the tissue contrast has improved with subsequent rehydration and restaining in aqueous iodine (Lugol's or IKI). In the present study this procedure resulted in superior differentiation of muscular and other soft tissues, whereas IKI staining resulted in very similar absorption values for muscle and cuticle, making the segmentation of structures of interest in these type specimens difficult. This was not the case in the study of the millipede species *Ommatoiulus avatar* [1], which showed good differentiation of structures with only I2E staining. For irreplaceable museum specimens—particularly type material—it is desirable to remove any X-ray contrast stain applied for imaging [1]. Iodine staining generally washes out in 70% ethanol [23, 24], the standard preservative for museum arthropod specimens. Other de-staining methods have been reported and merit further investigation.

### Modern systematic approaches and myriapod avatars

The increasing awareness of the global decline in biodiversity has generated incessant debates in the past few years on the inevitably growing extinction rates that imminently challenges the exercise of assessing the biological diversity on Earth ([25], [26], [27], [28]). Catalysed by the rapid development of new computational methods and next generation molecular and imaging technologies, several approaches have recently emerged to accelerate the pace of documenting and describing new taxa before they go extinct.

We agree about the need for efficient and quick methods for describing species (which can sometimes even be named from photos [29]), yet we advocate that integrative and multidisciplinary research may represent one of the most sustainable approaches linking various disciplines and offering a large spectrum of useful information for different fields of biosystematics. The 3D image data of the millipede *Ommatoiulus avatar* [1] were for example recently retrieved and used in a study on the taxonomic value of the vulva in millipedes of the family Julidae [30] and on the evaluation of the musculo-skeletal system of the diplopod head [31]. The 3D images of the millipede avatar and the cybercentipedes are also being used for several multidisciplinary ongoing research and educational purposes, which builds further awareness on the importance of the invertebrate fauna.

The 3D imaging in this study has confirmed a number of characters noted in the species description [9], allowed an in-depth examination of the male internal features and has unravelled further significant structures and allowed us to interpret some of their functionalities. The efficacy of microCT as a new tool in modern taxonomy is supported, the notion of cybertypes expanded, and the importance of volumetric image datasets of an actual type specimen demonstrated [32]. The ‘cybertype’ concept is not recognised by the ICZN (International code of Zoological nomenclature) as an established status *per se*. However, we take the occasion here to stress once more its importance as a complementary source of a wealth of data on type material–a virtual ‘type’ that transcends restrictions and limitations on obtaining valuable and often inaccessible physical type specimens. MicroCT image data provide details of the internal structures of intact specimens in a way that no other imaging method can. However, like all image-based data, it requires corroboration and complementation from conventional and broadly used imaging methods of the physical specimen, e.g. light microphotography and scanning electron microscopy [33], especially for surface structures (e.g., plectrotaxy, colour, etc.). Actual physical types can in most cases be returned undamaged after microCT imaging, and the cybertypes will further provide complementary information on internal structures and tissues that could be retrieved at any time from any corner of the world. Natural history museums worldwide are facing the challenge to document and digitize their physical type collections, and accurate volumetric data can certainly constitute another significant component in this, opening a new dimension in recording and sharing valuable and rare museum samples.

## Supporting information

S1 Video Volume rendering of *Eupolybothrus liburnicus*, male holotype CHP 545. Rotation along body axis.
(MPG)

S2 Video Volume rendering of *Eupolybothrus liburnicus*, female paratype CHP 583. Rotation along body axis and lateral virtual sectioning with highlighted eggs (orange).
(MP4)

S3 Video Volume rendering of *Eupolybothrus liburnicus*, male holotype CHP 545, showing intermediate tergite and prefemora of legs 15 with highlighted right prefemoral knob (cyan). (MP4)
